# Filarial Antigenemia and *Loa loa* Night Blood Microfilaremia in an Area Without Bancroftian Filariasis in the Democratic Republic of Congo

**DOI:** 10.4269/ajtmh.14-0358

**Published:** 2014-12-03

**Authors:** Didier K. Bakajika, Maurice M. Nigo, Jean Pierre Lotsima, Germain A. Masikini, Kerstin Fischer, Melanie M. Lloyd, Gary J. Weil, Peter U. Fischer

**Affiliations:** Programme National de Lutte contre L'Onchocercose, Kinshasa, Democratic Republic of Congo; Centre de Recherche en Maladies Tropicales de l'Ituri, Ituri, Congo, Democratic Republic of Congo; Infectious Diseases Division, Department of Internal Medicine, Washington University School of Medicine, St. Louis, Missouri

## Abstract

Implementation of mass drug administration for lymphatic filariasis (LF) has been delayed in central Africa because of incomplete mapping and coendemic loiasis. We mapped two regions in eastern Democratic Republic of Congo that were suspected to have LF. Night blood samples were collected from 2,724 subjects in 30 villages. Filarial antigenemia rates by card test exceeded 1% in 28 villages (range = 0–14%). Prevalence rates for large sheathed microfilariae (Mf) ranged from 4% to 40%; *Mansonella perstans* rates ranged from 22% to 98%. Large Mf were exclusively *Loa loa* by microscopy, and only 1 of 337 samples tested by quantitative polymerase chain reaction (qPCR) was positive for *Wuchereria bancrofti* DNA. Filarial antigen positivity was strongly associated with high *L. loa* Mf counts. Periodicity studies revealed atypical patterns, with no significant diurnal periodicity in some individuals. Thus, methods routinely used for LF mapping may not be reliable in areas in central Africa that are highly endemic for loiasis.

## Introduction

Lymphatic filariasis (LF) affects some 120 million people in 73 countries, and approximately 1.3 billion people are at risk of becoming infected with the nematode parasites (*Wuchereria bancrofti* and *Brugia* species) that cause this disease.^1^ The Global Program to Eliminate Lymphatic Filariasis (GPELF) is using mass drug administration (MDA) to reduce filarial infection rates below those required for sustained transmission with the goal of permanently eliminating LF in all endemic countries by the year 2020. The progress of GPELF is variable: some countries have already approached the elimination target, whereas others have not even started with MDA. Accurate mapping of the distribution of LF is a crucial first step for LF elimination programs. This is especially important for regions with loiasis, because ivermectin used in LF elimination programs can cause serious adverse events (including death) in persons with heavy *Loa loa* infections. Two tests that are widely used for mapping LF are microfilaria (Mf) detection (by microscopic examination of stained thick smears prepared with blood collected at night) and detection of filarial antigenemia (immunological detection of soluble *W. bancrofti* antigens in peripheral blood) by immunochromatographic card test (ICT). Because *W. bancrofti* Mf in Africa exhibit nocturnal periodicity and because *L. loa* Mf exhibit diurnal periodicity, large sheathed Mf in night blood are generally assumed to be *W. bancrofti*, and Mf present in blood collected during the day are assumed to be *L. loa*.^2^ *Mansonella perstans* is a third filarial species that infects humans in many areas of Africa, but *M. perstans* Mf can easily be distinguished from those of *L. loa* and *W. bancrofti* based on their smaller size and lack of a sheath.

The circulating *W. bancrofti* antigen that is detected by the ICT card test is present in blood collected during the day or night. For convenience reasons, the test is often performed with blood samples collected during the day. Furthermore, this test has be extensively evaluated and used in many parts of the world for mapping and monitoring LF elimination programs. However, the ICT card test has not been widely used in the past in central Africa, because large-scale LF elimination programs have not been started in most countries in this region. This paper shows that tests that are routinely used to map LF may not provide accurate results in areas of central Africa that are highly endemic for loiasis.

## Materials and Methods

### Study area.

**Figure 1.**
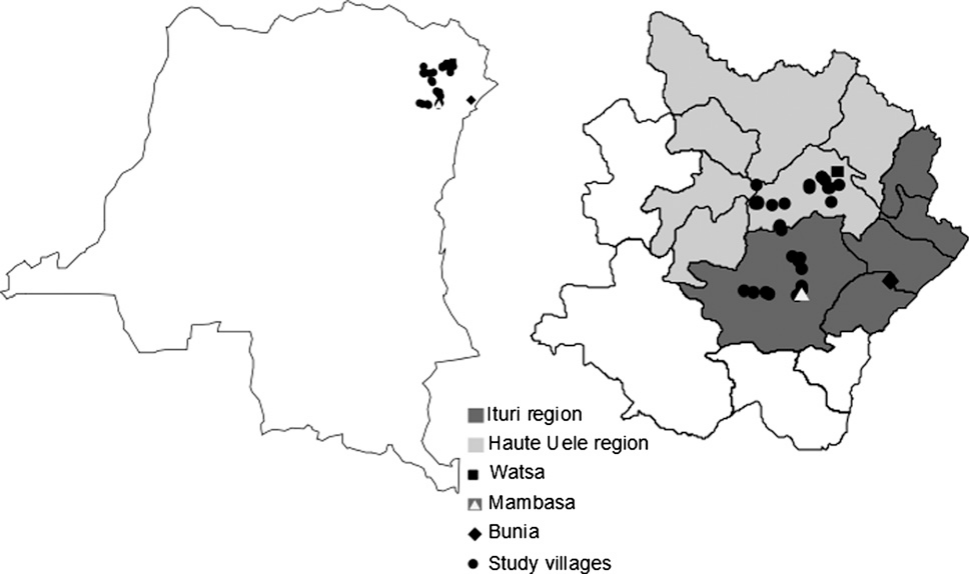
Map of (left panel) the study area in the northeastern DRC showing (right panel) the examined villages in the Mambasa and Watsa Territories.

In total, 14 villages in the Ituri region (Mambasa Territory) and 16 villages in the Haut Uele region (Watsa Territory) of the Orientale Province in the eastern Democratic Republic of Congo (DRC) (Figure 1 and Supplemental Table 1) were screened for Mf and filarial antigenemia. Most villages were located in remote forested areas east of the Okapi game reserve. The surveys were conducted in July of 2011 and January of 2013. No community-directed treatment with ivermectin had been performed in the study areas before these surveys. Historically the Ituri region has been reported to be endemic for *L. loa*, *W. bancrofti*, and *M. perstans* (with Mf in the blood) as well as *Onchocerca volvulus* and *M. streptocerca* (with Mf in skin).^3^ More recent rapid epidemiological mapping of onchocerciasis indicated mostly meso- and hyperendemic villages in Mambasa and lower endemicity in Watsa.^4^

### Sample collection.

A convenience sample of 50–100 individuals ages ≥ 14 years old was tested in each village between 21:00 and 01:00 hours to assess filarial antigenemia and Mf rates; 200 μL finger-prick blood was collected in (ethylenedinitrilo)tetraacetic acid (EDTA) -coated tubes.

### Antigen and Mf testing.

**Figure 2.**
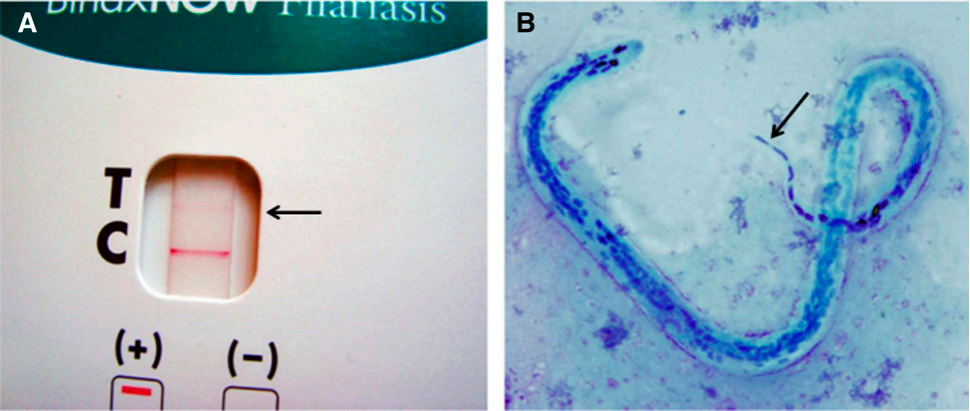
**A** shows a weakly positive ICT card test from a subject from the Ituri region with *L. loa* Mf. Note that the test line (T; marked with an arrow) is the same color as the control (C) line. **B** shows a Giemsa-stained *L. loa* Mf in a night blood smear from the same subject. Note the characteristic elongated nuclei in the tip of the tail (arrow).

The ICT test for circulating *W. bancrofti* antigen (Binax Filariasis Now Card Test; Alere, Scarborough, ME) was performed according to the manufacturer's instructions and read strictly at 10 minutes after applying the blood sample. Positive results were documented by photography (Figure 2). Mf testing was performed using three-line blood smears (60 μL blood total on the slide) as previously described.^5^ Giemsa-stained slides were read at 400× magnification, and numbers of small/thin (*M. perstans*) and large (*L. loa* or *W. bancrofti*) Mf were recorded. For each slide positive for large Mf, 10 large Mf were examined at a 1,000× magnification for differentiation of *L. loa* and *W. bancrofti*. Mf with a single nucleus in the tip of the tail were identified as *L. loa*. Slides positive for Mf were sent to Washington University in St. Louis, MO for reexamination by microscopy and DNA testing. Some positive and negative ICT cards were also sent to Washington University for DNA testing.

### Periodicity of *L. loa*.

The periodicity of *L. loa* Mf was studied in seven adults (four men and three women; age range = 28–78 years) in July of 2012. These individuals had been noted to have high *L. loa* Mf counts (> 2,000/mL) in night blood in 2011. Periodicity was assessed by counting Mf in 60-μL thick smears prepared with blood collected by finger prick at 05:00, 13:00, and 21:00 hours.

### Differentiation of *L. loa* and *W. bancrofti* by quantitative polymerase chain reaction.

High Mf densities in many slides made it impossible to examine all large Mf by microscopy at 1,000× magnification to exclude the presence of *W. bancrofti* Mf. Therefore, we used probe-based quantitative real-time polymerase chain reaction (qPCR) assays to detect filarial DNA. DNA extraction was performed 3–6 months after preparation of the Giemsa-stained blood smears. Sterile razor blades were used to scrape dried blood from stained blood smears (two lines; equivalent to 40 μL). DNA was isolated from dried blood using the QIAamp DNA Extraction Kit for Blood (QIAGEN, Valencia, CA). Alternatively, DNA was extracted from dried blood on one-half of an ICT card sample application pad as previously described.^6^ Blood samples were tested with two separate qPCR reactions for detection of W. *bancrofti* and *L. loa* DNA as previously described.^6^,^7^ Giemsa-stained slides from Côte d'Ivoire that contained *W. bancrofti* Mf but no *L. loa* Mf were used as positive controls for the *W. bancrofti* qPCR assay and negative controls for the *L. loa* qPCR assay.

### Statistics.

R 3.0.1 (http://www.r-project.org/)^8^ was used to develop the generalized linear model (GLM)^9^ and design the map.^10^,^11^ A GLM with a binomial function was developed to identify significant predictors of positive ICT test results. Predictor variables in this model included age, sex, number of *L. loa* Mf per milliliter, and number of *M. perstans* Mf per milliliter. Maps were drawn using the R3.0.1 packages Maps (http://cran.r-project.org/web/packages/maps/index.html) and Mapdata (http://cran.r-project.org/web/packages/mapdata/index.html).^10^,^11^ Global positioning system (GPS) coordinates of the villages were recorded during the study period, and data detailing the administrative areas were taken from DIVA-GIS (www.diva-gis.org).

### Ethical approval.

This mapping was performed as part of the National Program to Eliminate LF in the DRC. Collection of blood samples for the periodicity study was approved by the Ethical Committee of the School of Public Health, Kinshasa University, DRC.

## Results

### Prevalence rates for positive ICT tests and Mf.

In total, 2,724 individuals were tested in 30 villages in the Ituri and Haut Uele regions. Overall prevalence rates for filarial antigenemia and Mf in night blood with large sheathed Mf were 6% and 22%, respectively (Table 1). Positive ICT tests were observed in 28 of 30 villages, and 7 villages had ICT rates of at least 10%. Positive ICT tests had clearly visible T lines that were the same color as the control or C lines (Figure 2A). This result distinguishes these test results from false-positive tests that occur when tests are read well after the recommended read time of 10 minutes.^12^

Prevalence rates by village for large sheathed Mf in night blood ranged between 4% and 40% (Table 1). Geometric mean Mf densities in infected individuals ranged from village to village from 92.6 to 567.7 Mf/mL, and some individuals had Mf counts of > 20,000/mL. The maximum number of large Mf of 47,448 Mf/mL was detected in an 85-year-old woman who had lived in Apodo for the last 35 years. She also had *M. perstans* (1,549 Mf/mL). Many of the large sheathed Mf were unambiguously identified as *L. loa* by morphology (Figure 2B), and no *W. bancrofti* Mf were identified. However, given the high number of large Mf in the slides (12% of the samples with large Mf had > 120 Mf per 60 μL), it was impossible to exclude the presence of W. *bancrofti* Mf by microscopy alone.

*M. perstans* Mf are much smaller than Mf of *L. loa* or *W. bancrofti*, and they were easily differentiated by microscopy. *M. perstans* prevalence rates by village ranged from 22% to 98%. Eighteen (60%) of the villages surveyed had *M. perstans* Mf prevalence rates of at least 70%. Geometric mean Mf counts for individuals with *M. perstans* by village ranged between 221.6 and 2,605.3 Mf/mL, with a geometric mean density of 582.3 Mf/mL. This number is more than two times as high as the mean density of *L. loa* Mf.

### Species identification of Mf in night blood by qPCR.

qPCR testing was performed on blood from 337 slides that were positive for large sheathed Mf by microscopy (Table 2). *L. loa* DNA was detected in 294 (87%) samples. Because some blood samples contained very few Mf and recovery of DNA from stained smears is incomplete, it is likely that all tested slides with large Mf contained *L. loa* DNA. In contrast, *W. bancrofti* DNA was detected in only 1 of 337 blood samples tested (0.3%). This sample was from a 66-year-old woman who had lived in Ekwe village for the past 5 years and also had loiasis. We do not have information on where she lived before that time. We also tested sample application pads from 52 positive and 60 negative ICT cards for the presence of *L. loa* and *W. bancrofti* DNA; 36 of 52 subjects with positive ICT card results had large sheathed Mf in their thick blood smears, and 33 of the sample application pads from these ICT cards were positive for *L. loa* DNA by qPCR. Blood from the same female patient mentioned above from Ekwe was positive for DNA of both *L. loa* and *W. bancrofti*, but none of the other 111 card test application pads were positive for *W. bancrofti* DNA; 1 of 16 ICT positive blood samples from people with blood smears that did not contain large Mf was positive for *L. loa* DNA by qPCR. Also, 10 of 60 ICT negative card tests were from people with large Mf in night blood smears, and 8 of them were positive for *L. loa* DNA by qPCR. The other 50 ICT negative samples had no large Mf in night blood, and 1 of these samples was positive for *L. loa* DNA by qPCR. These results show that (with one exception) the large Mf present in night blood samples collected in this study were *L. loa*.

### Relationship between ICT card positivity and *L. loa* Mf density.

ICT card test positivity was clearly linked to the presence and number of *L. loa* Mf in night blood smears (Table 3). In total, 131 of 577 (22.7%) blood samples from people with *L. loa* Mf had positive ICT tests. Although only 2% of individuals without *L. loa* Mf in night blood had positive antigen tests, almost 60% of those with Mf counts > 2,000/mL had positive ICT tests; 7 of 15 subjects with Mf counts between 8,000 and 30,000 Mf/mL had positive ICT tests. The only person with > 30,000 Mf/mL in the night blood was the elderly woman mentioned above who had a negative ICT test.

The ICT positivity rate for blood samples from persons who were negative for *M. perstans* Mf and persons with low (≤ 100 Mf/mL), medium (101–1,999 Mf/mL), or high *M. perstans* Mf counts (> 2,000 Mf/mL) ranged between 4% and 10%. Mf counts between 8,000 and 30,000 Mf/mL were detected in 65 individuals, and only 8 of these people had positive ICT test results. Higher *M. perstans* Mf counts were observed in only four subjects, and all of these subjects had negative ICT test results. A GLM comparing the differences in ICT test results with *M. perstans* Mf counts found no relationship between these two variables, whereas high *L. loa* Mf counts were strongly associated with positive ICT test results (Table 4).

### Periodicity of *L. loa* Mf.

**Figure 3.**
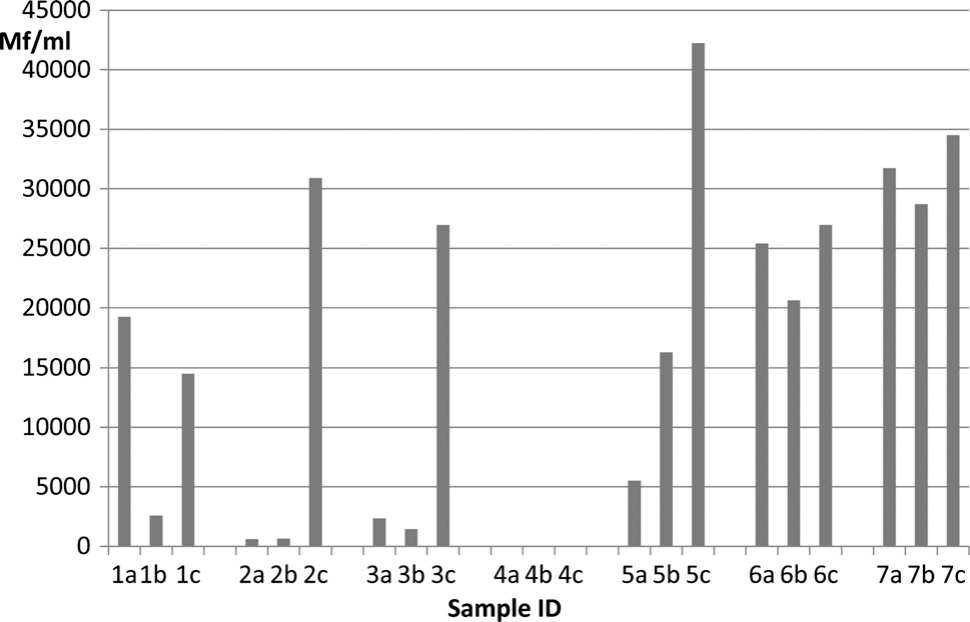
Periodicity of *L. loa* Mf in seven patients with *L. loa* Mf in night blood smears. One subject (subject 4) had no Mf at the time of reexamination. The others had variable periodicity patterns that were diurnal (subjects 2 and 3), diurnally subperiodic (subject 5), and aperiodic (subjects 6 and 7); 60-μL finger prick blood samples were collected at (a) 21:00, (b) 05:00, and (c) 13:00 hours, and Mf densities were determined by microscopy. The species identification was confirmed to be *L. loa* in all cases by qPCR.

Periodicity studies were performed because of the unexpected finding that *L. loa* Mf were commonly seen in night blood. Finger prick blood was collected at 21:00, 05:00, and 13:00 hours from seven people that had *L. loa* Mf present in night blood samples during the first survey in 2011. All of these individuals were coinfected with *M. perstans* at that time, with Mf densities between 333 and 8,413 Mf/mL. At the time of reexamination in 2012, one man was Mf-negative, and the others had night blood *L. loa* Mf counts between 608 and 31,728 Mf/mL. Two individuals (subjects 2 and 3) had a diurnally periodic pattern, with almost no Mf at 21:00 and 05:00 hours but high counts of > 25,000 Mf/mL at 13:00 hours (Figure 3). Subjects 1 and 5 had diurnally subperiodic patterns, and two individuals (subjects 6 and 7) had aperiodic patterns, with > 20,000 Mf/mL at all three time points. These results show that *L. loa* exhibits atypical periodicity in some subjects in the study area, and many subjects had high Mf counts in night blood.

## Discussion

The purpose of this study was to map the distribution of LF in the Ituri and Haut Uele regions of the DRC before initiation of MDA for LF elimination. With only 1 *W. bancrofti* infection identified (by PCR only) from > 2,700 people tested, it is safe to say that MDA is not required for these regions. This finding underlines the importance of careful mapping before initiating MDA for LF in central Africa, where the infection is often highly focal and historical information on prevalence is spotty.

In contrast to *W. bancrofti*, infections with *L. loa* and *M. perstans* were highly prevalent in the study areas. The presence of loiasis was not surprising, because recent rapid assessment of prevalence of *Loa loa* (RAPLOA) surveys had documented high rates of loiasis in these regions.^13^,^14^ However, high rates of *L. loa* Mf in night blood samples in this study were a major surprise. This result contrasts with the common assumption that people in central Africa with large sheathed Mf in night blood have bancroftian filariasis, whereas those with large sheathed Mf in day blood have loiasis.^2^ Periodicity studies showed that *L. loa* Mf counts did not exhibit the expected diurnal pattern in some subjects in the study areas. Additional research is needed to determine the relative frequency of these atypical periodicity patterns (diurnal subperiodic and aperiodic) and assess whether particular genotypes are associated with aperiodic *L. loa*.

The presence of high numbers of *L. loa* Mf in night blood samples has important implications for mapping LF in areas of central Africa with loiasis. It is not possible to rule out the presence of *W. bancrofti* Mf by microscopy when *L. loa* Mf counts are high in night blood samples. Although we assumed that this problem could be avoided by antigen testing, our results suggest that the ICT test may not be specific for *W. bancrofti* infection in loiasis coendemic areas of Africa. Prior studies have shown that, although the epitope recognized by the monoclonal antibody AD12.1 is present in antigen extracts from many nematode species, filarial antigen tests, such as the Binax Now Filariasis Test, that use this antibody (or the closely related Og4C3 monoclonal antibody) have until now been considered to be specific for *W. bancrofti* infection. Prior studies of the Binax Now Filariasis Test have not detected filarial antigen in serum samples from patients infected with *B. malayi*, *O. volvulus*, *M. perstans*, or *L. loa* (apart from a few samples from people who had also been exposed to *W. bancrofti*).^15^–^17^ The ICT card test has been extensively used in areas with onchocerciasis, and no evidence for cross-reactivity has been observed.^18^,^19^ However, relatively few serum or plasma samples from patients with loiasis were tested in these prior studies, and some of these samples were from Americans who had acquired the infection as missionaries or Peace Corps volunteers in Africa. It seems likely that some people with loiasis in the study area have a circulating *L. loa* antigen that is immunologically cross-reactive with the 200-kDa *W. bancrofti* adult worm antigen that is detected by the Binax Now Filariasis ICT Test.^16^ Because most (77%) blood samples from subjects with *L. loa* Mf had negative ICT test results, ICT positivity in loiasis is likely to be associated with high *L. loa* adult worm loads. Prior studies have shown that circulating filarial antigen levels are correlated with adult female worm counts in dogs infected with *Dirofilaria immitis* and jirds infected with *B. pahangi*.^20^,^21^ An association with adult worm loads would explain the increased frequency of ICT positivity in subjects with very high night blood Mf counts and the lack of positivity in prior studies with loiasis serum from expatriates and Africans from other *Loa*-endemic countries. The relatively high frequency of negative ICT tests in people with Loa Mf counts > 2,000/mL (41%) might be explained by immune clearance of the putative circulating *L. loa* antigen in some subjects.

Because day blood Mf counts were not performed in this study, we do not know whether the ICT test might be useful in areas without LF for detecting persons with very high *L. loa* Mf counts (> 30,000/mL) who have an increased risk of developing neurological severe adverse events after ivermectin.^22^ Additional studies are needed (with day blood testing in areas with high rates of loiasis) to answer this question.

The specificity problem that we observed with the ICT card test in central Africa may be even worse with the Alere Filariasis Test Strip, which also uses the AD12.1 monoclonal antibody, because the Test Strip is more sensitive than the ICT card test.^15^ It has to be stressed that positive ICT results caused by cross-reactivity of a *Loa* antigen are true-positive results that can be confirmed by retesting. These results are different from false-positive ICT tests caused by improper reading of the diagnostic device that are not confirmed when tests are read correctly after 10 minutes. This finding leads to the question of how best to map and monitor LF in areas with high rates of loiasis. Traditional night blood Mf testing might be sufficient for this purpose in areas where *L. loa* Mf have strictly diurnal periodicity. Antibody testing with the recombinant filarial antigen Wb-123 could be helpful, but there is no commercially available version of the test at this time.^23^ Similar to the situation with the ICT test, the specificity of Wb-123 antibodies for *W. bancrofti* infection has not yet been thoroughly evaluated with serum or plasma samples from *Loa*-endemic areas in central Africa. For now, we think that the best option for mapping LF in *Loa*-endemic regions would be to test night blood samples for *W. bancrofti* DNA by qPCR.^24^ Dried blood samples can be pooled to reduce costs, and night blood surveys should be powered to provide high confidence for detecting *W. bancrofti* infection rates that exceed 1% in implementation units, because that is the trip point for initiating MDA. Several reference laboratories in Africa have experience with qPCR testing for LF.

In conclusion, this study did not detect a significant presence of LF in Mambasa or Watsa Territories, and MDA is not required for that infection in these areas. However, the study identified factors that will complicate mapping and monitoring activities for LF elimination programs in areas of central Africa that are highly endemic for loiasis. They include high rates of *L. loa* Mf in night blood samples that interfere with detection of *W. bancrofti* Mf by microscopy and the unexpected finding that some subjects with high-intensity *L. loa* infections have positive filarial antigen tests. LF elimination programs in areas of central Africa with high rates of loiasis may need to use alternative diagnostic tools, such as antibody tests or qPCR, for mapping, monitoring, and evaluation.

## Supplementary Material

Supplemental Table.

## Figures and Tables

**Table 1:** Summary of filarial antigenemia and Mf test results from night blood surveys in the Ituri and Haut Uele regions in the eastern DRC by village

Village name	*N* tested	ICT positive (%)	Rate of *L. loa* Mf (%)	Geometric mean* of *L. loa* (Mf/mL)	Rate of *Mp* Mf (%)	Geometric mean* of *Mp* Mf (Mf/mL)
Ituri						
Memekidele	71	10	27	160.3	97	2,605.3
Aluta	100	10	19	241.2	98	547.7
KeroZanzibar	90	7	21	168.1	85	877.4
Digbo	77	6	27	325.4	97	2,033.9
Ekwe	100	5	13	342.1	94	1,481.3
Epulu	98	5	17	155.6	44	379.3
Salate	89	4	10	567.7	62	413.4
Saiyo	53	4	19	545.0	83	2,018.0
Nduye	100	3	26	257.8	78	276.7
Komboni	100	3	21	309.5	92	1,194.1
Molokayi	100	3	10	302.2	61	449.9
Malembi	100	2	13	290.1	96	1,048.9
Bapukeli	98	2	19	147.2	70	913.4
Butiaba 2	50	0	16	180.5	86	1,125.5
Haut Uele						
Bayitebi	100	14	37	150.3	76	410.5
Obo II	100	13	30	161.3	75	221.6
Kossia	100	12	29	203.4	79	648.6
Obo I	100	10	37	314.1	76	288.1
Luwi	100	10	27	285.6	79	547.8
Apodo	100	8	40	451.2	73	429.7
Netiti-Gombari	100	8	25	278.1	67	562.3
Tibodri	100	8	26	137.8	65	506.9
Bakiri	100	7	35	267.3	59	832.8
Dodi	100	7	14	270.7	91	1,205.5
Andekofu	88	6	20	324.1	61	499.1
Osso I	100	5	24	212.5	63	607.1
Kadungu	87	3	21	109.1	50	351.1
Ngili-ngili	94	2	4	139.9	22	616.8
Andra	76	1	20	113.8	30	1,367.3
Toli	53	0	11	92.6	43	594.9
Total	2,724	6	22	231.2	72	582.3

Large sheathed Mf were identified by morphology as *L. loa*, whereas small unsheathed Mf were identified as *M. perstans* (*Mp*). Mf densities were not available for all *Mp*-positive samples from some villages.

* The geometric mean number of Mf was calculated for Mf-positive subjects.

**Table 2:** Detection of filarial antigenemia (ICT) and parasite DNA in night blood samples from subjects with large sheathed Mf in night blood smears

Village	*N* tested by qPCR		*N* positive by *L. loa* DNA by qPCR	*N* positive for *W. bancrofti* DNA by qPCR
	ICT positive	ICT negative		
Ituri				
Memekidele	5	15	19	0
Aluta	8	12	18	0
KeroZanzibar	3	16	18	0
Digbo	4	16	12	0
Ekwe	4	9	12	1
Epulu	1	16	14	0
Salate	2	7	8	0
Saiyo	2	8	10	0
Nduye	3	23	23	0
Komboni	4	18	17	0
Molokayi	2	8	9	0
Malembi	3	11	13	0
Bapukeli	1	18	16	0
Butiaba 2	0	8	8	0
Haut Uele				
Bayitebi	14	0	13	0
Obo II	13	0	11	0
Kossia	12	0	8	0
Obo I	10	0	9	0
Luwi	10	0	7	0
Apodo	8	3	11	0
Netiti-Gombari	7	0	7	0
Tibodri	6	0	6	0
Bakiri	7	0	7	0
Dodi	7	0	7	0
Andekofu	4	0	4	0
Osso I	5	2	5	0
Kadungu	2	0	2	0
Ngili-ngili	0	0	–	–
Andra	0	0	–	–
Toli	0	0	–	–
Total	147	190	294	1

**Table 3:** Relation of positive filarial antigen test results (ICT) with *L. loa* and *M. perstans* Mf count

Mf density (Mf/mL)	ICT negative		ICT positive		ICT invalid		Total *N*
	*N*	Percent	*N*	Percent	*N*	Percent	
*L. loa*							
0	2,065	97.59	37	1.75	14	0.66	2,116
1–100	206	91.56	18	8.00	1	0.44	225
101–2,000	240	76.68	72	23.00	1	0.32	313
> 2,000	29	41.43	41	58.57	0	0	70
Total	2,540	93.25	168	6.17	16	0.58	2,724
*M. perstans*							
0	673	95.33	29	4.10	4	0.57	706
1–100	270	94.07	17	5.92	0	0	287
101–2,000	740	90.13	77	9.38	4	0.49	821
> 2,000	282	88.96	33	10.41	2	0.63	317
Total	1,965*	92.21	156*	7.32	10*	0.47	2,131*

Mf were counted on a single 60-μL night blood smear, and counts were converted to Mf per milliliter.

* *M. perstans* Mf counts were not available for all *M. perstans* Mf-positive individuals. Slides from 593 *M. perstans* Mf-positive and *L. loa* Mf-negative subjects from six villages were not counted.

**Table 4:** Results from a GLM that assessed associations between filarial antigen test results and age, sex, and Mf counts for *L. loa* and *M. perstans*

Variable	Estimate	SE	*Z* score	*P* value
Intercept	−3.176	1.043	−11.208	< 0.0001
Age	0.174	0.302	2.114	0.0345
Sex (males)	0.697	0.766	0.910	0.363
*L. loa* (Mf/mL)	0.089	0.066	4.958	< 0.0001
*M. perstans* (Mf/mL)	−0.045	0.036	−1.276	0.202
