# Supplementary material for: Filarial Antigenemia and Loa loa Night Blood Microfilaremia in an Area Without Bancroftian Filariasis in the Democratic Republic of Congo
Source: Am J Trop Med Hyg. 2014 Dec 3;91(6):1142–8. doi: 10.4269/ajtmh.14-0358 (PMC4257636; doi:10.4269/ajtmh.14-0358)
Supplement: Supplementary file 1 [file SD2.pdf]

Supplemental Table 1

Geographic coordinates for villages examined for filarial antigenemia and night blood microfilaremia in the Ituri and Haut Uele regions of the northeastern Democratic Republic of Congo

Region/village name	Longitude	Latitude	Elevation (m)
Ituri/Mambasa			
Memekidele	E 29°01’24.7”	N 01°52’12.7”	658
Aluta	E 29°03’26.2”	N 01°43’32.0”	895
KeroZanzibar	E 29°02’36.2”	N 01°25’45.2”	866
Digbo	E 28°44’58.3′’	N 02°18’59.2”	826
Ekwe	E 29°02’58.0”	N 01°27’23.3′’	858
Epulu	E 28°34’19.3′’	N 01°24’13.1”	754
Salate	E 28°23’43.0”	N 01°24’12.9”	739
Saiyo	E 28° 58’ 56.4”	N 01°22’05.8”	837
Nduye	E 28°59’58.2”	N 01°49’40.6”	823
Komboni	E 28°56’01.9”	N 01°53’26.3′’	828
Molokayi	E 28°16’52.5′’	N 01°26’08.1”	686
Malembi	E 28°47’06.6”	N 02°15’12.8”	843
Bapukeli	E 28° 36’ 58.5′’	N 01°23’26.1”	791
Butiaba 2	E 29° 02’ 48.8”	N 01°19’40.3′’	913
Haut Uele/Watsa			
Bayitebi	E 28° 49’ 04.1”	N 02° 37’ 06.6”	858
Obo II	E 29° 10’ 00.9”	N 02° 51’ 28.9”	856
Kossia	E 28° 27’ 08.4”	N 02° 37’ 21.4”	819
Obo I	E 28° 26’ 08.6”	N 02° 51’ 29.7”	854
Luwi	E 29° 09’ 34.6”	N 02° 49’ 09.1”	843
Apodo	E 28° 38’ 45.5′’	N 02° 35’ 48.8”	851
Netiti-Gombari	E 29°27’ 08.5′’	N 02° 38’ 24.9”	859
Tibodri	E 28° 25’ 09.6”	N 02° 36’ 27.4”	876
Bakiri	E 28° 26’ 07.5′’	N 02° 38’ 23.9”	820
Dodi	E 28° 27’ 09.3′’	N 02° 36’ 27.7”	817
Andekofu	E 29° 20’ 02.00”	N 02° 58’ 43.1”	1,008
Osso I	E 29° 24’ 26.2”	N 02° 49’ 52.7”	946
Kadungu	E 29° 25’ 25.3′’	N 02° 49’ 53.8”	940
Ngili-ngili	E 29° 33 06.8”	N 02° 51’ 52.7”	983
Andra	E 29° 21’ 57.7”	N 02° 56’ 16.8”	915
Toli	E 29° 19’ 01.1”	N 02° 57’ 44.0”	1,012
